# Acid sphingomyelinase deficiency and Gaucher disease in adults: Similarities and differences in two macrophage storage disorders

**DOI:** 10.1002/jmd2.12420

**Published:** 2024-07-04

**Authors:** Eline C. B. Eskes, Laura van Dussen, Johannes M. F. G. Aerts, Martijn J. C. van der Lienden, Mario Maas, Erik M. Akkerman, André B. P. van Kuilenburg, Barbara Sjouke, Carla E. M. Hollak

**Affiliations:** ^1^ Endocrinology and Metabolism Amsterdam UMC location University of Amsterdam Amsterdam The Netherlands; ^2^ Amsterdam Gastroenterology Endocrinology Metabolism, Inborn Errors of Metabolism Amsterdam The Netherlands; ^3^ Department of Medical Biochemistry Leiden Institute of Chemistry, Department of Medical Biochemistry Leiden The Netherlands; ^4^ Radiology and Nuclear Medicine Amsterdam UMC location University of Amsterdam Amsterdam The Netherlands; ^5^ Laboratory Genetic Metabolic Diseases Amsterdam UMC location University of Amsterdam Amsterdam The Netherlands; ^6^ Department of Internal Medicine Radboud UMC Nijmegen The Netherlands

**Keywords:** acid sphingomyelinase deficiency, biomarkers, Gaucher disease, sphingolipids

## Abstract

The lysosomal storage diseases chronic visceral acid sphingomyelinase deficiency (ASMD) and Gaucher disease type 1 (GD1) are both macrophage storage disorders with overlapping clinical manifestations. We compared cross‐sectional data on visceral, hematological, and biochemical manifestations of untreated adult patients with chronic visceral ASMD (*n* = 19) and GD1 (*n* = 85). Spleen volume, liver volume, and bone marrow fat fraction did not significantly differ between the two disease groups (*p* >0.05 for all). Chitotriosidase activity was higher in GD1 (GD1: median 30 940 nmol/(mL.h), range 513–201 352, ASMD: median 1693 nmol/(mL.h), range 326–6620, *p* <0.001), whereas platelet levels were lower (GD1: median 102 10^9^/L, range 16–726, ASMD: median 154 10^9^/L, range 86–484, *p* <0.010), as were hemoglobin levels (GD1: median 7.8 mmol/L, range 5.0–10.4, ASMD: median 9.0 mmol/L, range 7.0–10.4, *p* <0.001). No bone complications were reported for ASMD, compared to 33% in GD1 (*p* <0.005). In ASMD pulmonary disease was more severe as evidenced by a median diffusion capacity of the lungs for carbon monoxide of 73% of predicted (range 26–104), compared to 85% (range 53–126) in GD1 (*p* = 0.029). In conclusion, bone complications, hematological abnormalities, chitotriosidase activity, and CCL18 levels were more prominent in GD1, while pulmonary manifestations were more common in AMSD. Different secondary pathophysiological processes surrounding sphingomyelin and glucosylceramide accumulation might explain these differences.

**Synopsis**

Bone complications, hematological abnormalitiesand elevation of biochemical markers are more excessive in untreated patients with Gaucher diseasetype 1 (GD1) as compared to untreated patients with the chronic visceral subtype of Acid Sphingomyelinase Deficiency (ASMD), while pulmonary manifestations are more common in ASMD. Differences between ASMD and GD1 might be explained by a higher sphingolipid load in GD1 and differences in the secondary pathophysiological processes surrounding sphingolipid accumulation in both diseases.

## 1 INTRODUCTION

Acid Sphingomyelinase Deficiency (ASMD, OMIM 607616), also known as Niemann‐Pick disease types A and B, and Gaucher disease type 1 (GD1, OMIM 230800) are lysosomal storage diseases (LSDs) with many similar disease manifestations. Both LSDs are characterized by sphingolipid accumulation. In case of ASMD sphingomyelin accumulates due to deficiency of acid sphingomyelinase (EC 3.1.4.12); whereas in GD1 the enzyme β‐glucosidase (EC 4.2.1.25) is deficient, resulting in accumulation of glucosylceramide.^1^, ^2^ In both LSDs, the accumulation of sphingolipids occurs mainly in macrophages.

**FIGURE 1:**
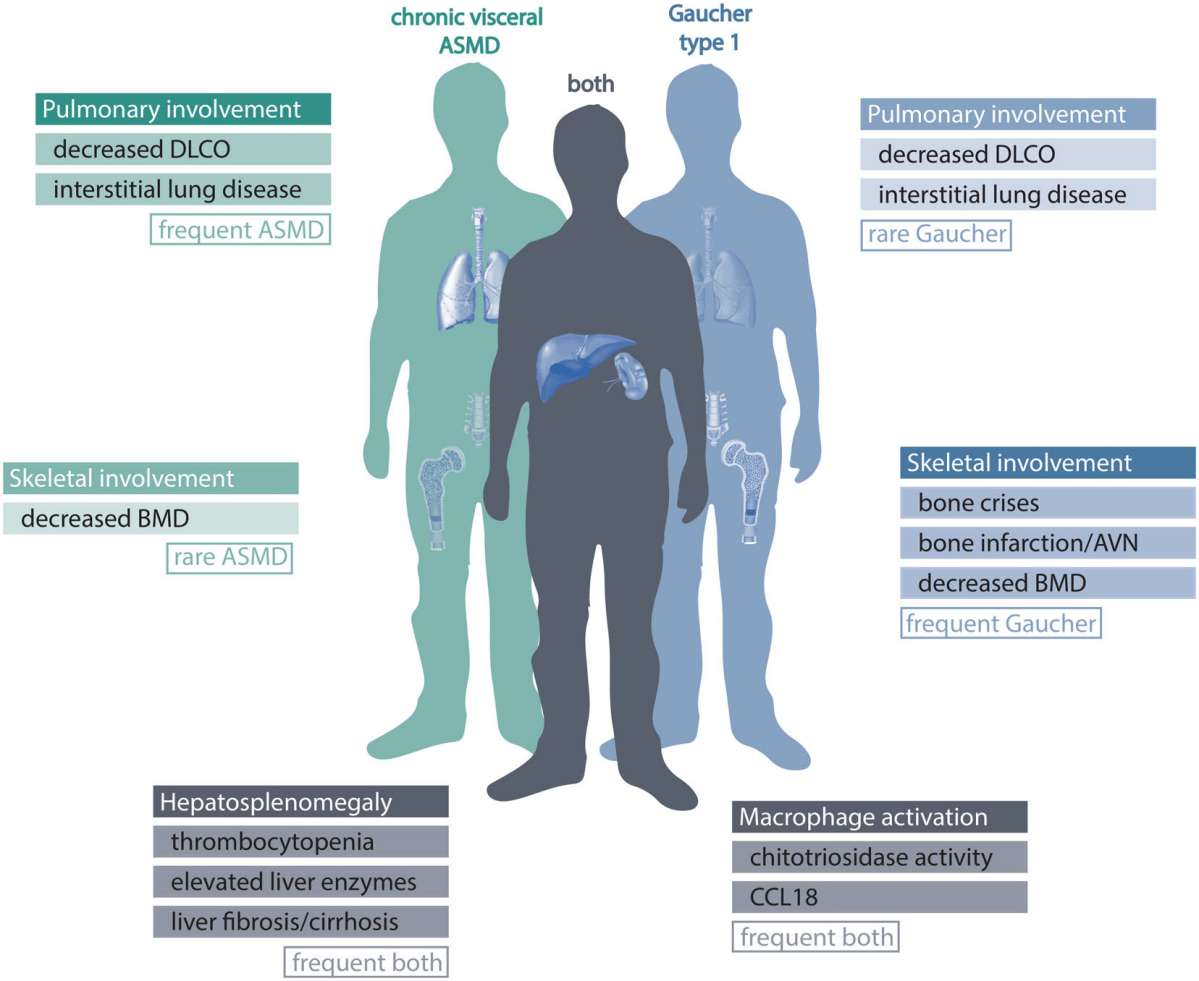
Clinical manifestations of ASMD and Gaucher, overlap and differences. ASMD, acid sphingomyelinase deficiency; AVN, avascular necrosis; BMD, bone mineral density; CCL18, chemokine C‐C motif ligand 18; DLCO, diffusion capacity of the lungs for carbon monoxide.

Both diseases cover a broad clinical spectrum, varying from children with severe neurological manifestations who die at a young age (i.e., the infantile neurovisceral subtype of ASMD and Gaucher disease type 2) to adult patients with mild symptoms restricted to visceral organs who show slow progression (i.e., the chronic visceral subtype of ASMD and GD1). Characteristic manifestations of both LSDs are splenomegaly, cytopenia, hepatomegaly, and elevated plasma levels of macrophage‐associated biomarkers (i.e., chitotriosidase activity and chemokine C–C motif ligand 18 (CCL18)) (see Figure 1).

Two therapeutic options to treat the visceral manifestations of GD1 are available within the EU: enzyme replacement therapy (ERT) and substrate reduction therapy.^3^, ^4^ Market authorization for ERT for the treatment of non‐central nervous system manifestations of ASMD (olipudase alfa, Sanofi Genzyme) has recently been granted by the European Medicines Agency and the Food and Drug Administration.^5^

Apart from similarities, differences between the clinical manifestations of these diseases exist. Pulmonary manifestations (i.e., interstitial lung disease and decreased diffusion capacity of the lungs for carbon monoxide (DLCO)) are present in a large part of ASMD patients^6^ and have only been described in GD1 in rare cases.^7^, ^8^ Severe bone marrow infiltration leading to complications such as infarctions and pathological fractions is common in GD1 and has a major impact on quality of life.^9^ Bone involvement has been occasionally described in ASMD patients and seems to be limited to decreased bone mineral content and density.^10^, ^11^

Insight into the similarities and differences between ASMD and GD1 could aid in understanding the differences in the occurrence and type of clinical manifestations. Biomarkers can be used as surrogate markers of clinical manifestations.^12^, ^13^ Because of the similarity in clinical manifestations and pathophysiology, markers that are used to assess disease in ASMD largely overlap the biomarkers used in GD1. Since GD1 has been studied more extensively, lessons could be learned for ASMD. Therefore the primary aim of this study was to compare markers of chronic visceral ASMD and GD1 in order to define the similarities and differences between both diseases. The secondary aim was to assess whether potential differences reflect a different prognostic value of markers regarding clinical manifestations in ASMD.

## 2 METHODS

### 2.1 Recruitment of patients

The Amsterdam UMC, location AMC is the national referral center for both ASMD and Gaucher disease in the Netherlands. GD1 patients have been monitored closely since the early 1990s and chronic visceral ASMD patients since 2005. All patients provided written consent for the collection of clinical data, which took place between January 1991 and March 2022. The study (2014_092#A201468) was approved by the local Medical Ethics Committee of the Amsterdam University Medical Centers, location Academic Medical Center.

### 2.2 Data collection

Data were retrieved from our prospective database in which patients with a confirmed diagnosis of the chronic visceral subtype of ASMD or GD1 are enrolled. Only data from untreated adult patients were retrieved. Data were collected for one time point per patient. If not all measurements were performed at the same visit, measurements within a year were included. If there were multiple time points with all measurements available, the most recent time point was included.

ASMD patients were monitored according to the clinical pathway for GD1 patients, which was adjusted over time based on experience and scientific literature. All patients underwent periodical assessment of plasma markers (e.g., hematological markers and macrophage‐associated markers), and magnetic resonance imaging (MRI) of spleen, liver, and lumbar vertebrae. For the latter the quantitative chemical shift imaging (QCSI) technique was used to assess fat fraction. In GD1 this is a validated biomarker for bone involvement and general disease burden.^14^ In ASMD patients, pulmonary function tests were periodically performed. Pulmonary function tests were not standardly performed in GD1 patients, but have been done when patients expressed pulmonary complaints and in the context of a study.

### 2.3 Interpretation of data

For the plasma levels of hematological markers (i.e., hemoglobin, leucocytes, and platelets) local reference ranges were applied. Chitotriosidase activity in plasma and CCL18 plasma levels were measured as described previously.^15^, ^16^ For patients with the 24‐bp duplication on one allele of the *CHIT1* gene, chitotriosidase activity was multiplied by two.^17^ Patients with a chitotriosidase deficiency (i.e., homozygous for 24‐bp duplication in *CHIT1* gene) were excluded from the analyses where appropriate. Fat fractions and bone marrow burden (BMB) scores were measured with MRI‐QCSI as described by Maas et al.^18^, ^19^ BMB scores can vary from 0 to a maximum of 16.^19^

### 2.4 Statistical analyses

Data were analyzed using R studio version 4.0.3. Descriptive statistics include frequencies or median and range. To assess differences between two groups Mann Whitney U tests and Fisher's exact tests were used for respectively continuous and dichotomous data. The log‐rank test was used to compare the probability of bone complications against age between ASMD and GD1 patients. The Spearman correlation coefficient was calculated to establish correlations between two continuous variables. *P*‐values were considered statistically significant when 0.05 or lower.

## 3 RESULTS

**FIGURE 2:**
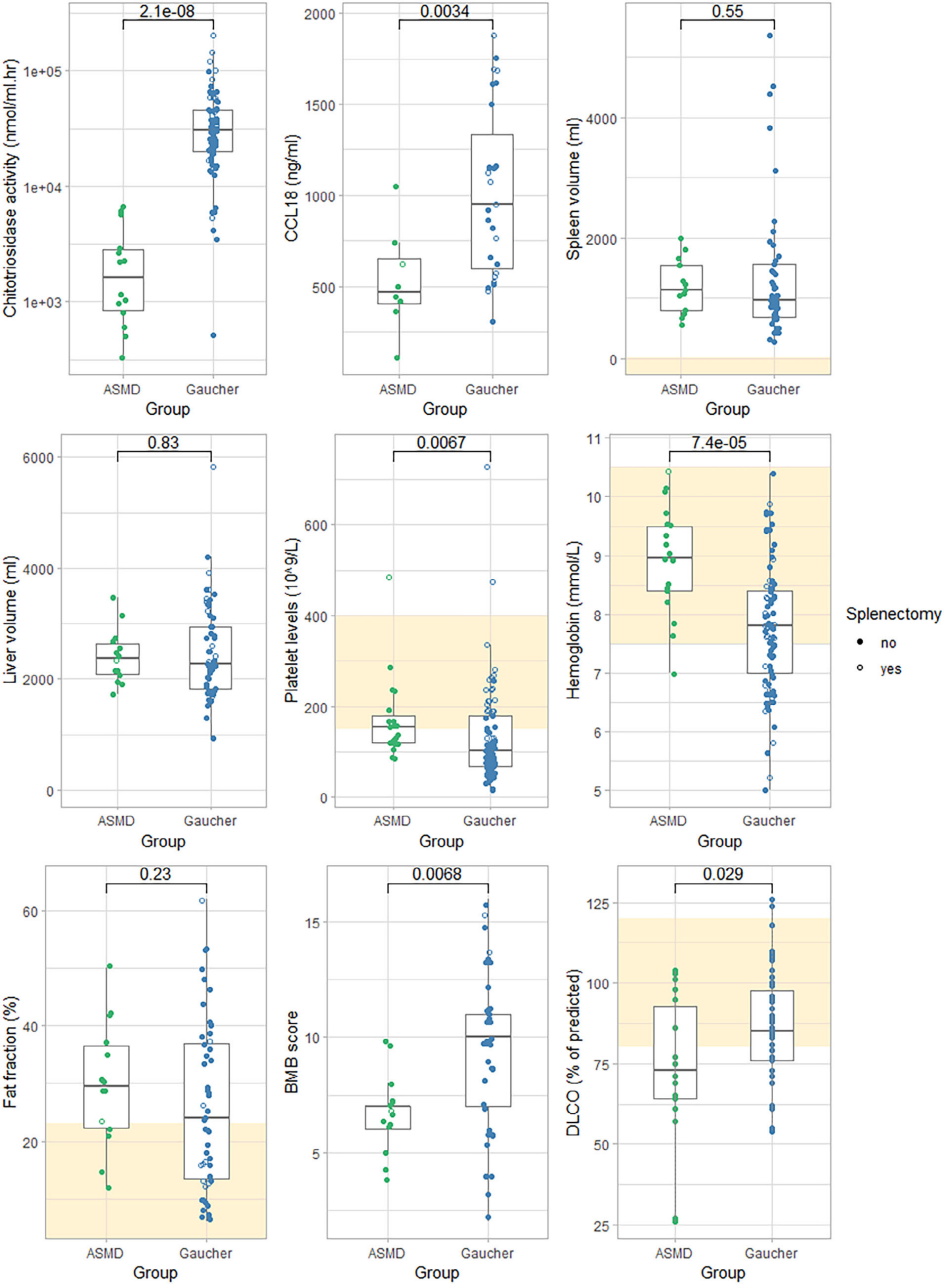
Hematological, biochemical and imaging markers of patients with ASMD compared to Gaucher patients. Boxes show median and interquartile ranges, yellow squares indicate reference ranges if available. ASMD, acid sphingomyelinase deficiency; CCL18, chemokine C–C motif ligand 18; BMB score, bone marrow burden score; DLCO, diffusion capacity of the lungs for carbon monoxide.

In this cross‐sectional cohort study, data of 19 adult ASMD patients with the chronic visceral subtype and 85 adult GD1 patients were included. At all included data points, patients were treatment‐naïve. Demographic characteristics and clinical manifestations are summarized in Table 1. Sex and age distribution of the two groups were similar, but a difference between the number of splenectomized patients in both groups was present (1/19 (5%) in ASMD and 26/85 (31%) in GD1, *p* = 0.022). For an overview of the markers discussed below, see Table 2 and Figure 2.

**TABLE 1:** Demographics and clinical manifestations.

	ASMD		Gaucher		*p*‐value
Number of patients	19		85		
Mutation					
R610del homozygous (%, *n*)	47%	9/19	‐		
R610del heterozygous (%, *n*)	5%	1/19	‐		
N370S homozygous (%, *n*)	‐		9%	8/85	
N370S heterozygous (%, *n*)	‐		76%	65/85	
Other (%, *n*)	47%	9/19	14%	12/85	
Age in years (median, range)	39.2 (18.7–64.2)		46.2 (17.6–65.4)		0.258
Female sex (*%, n*)	37%	7/19	45%	38/85	0.614
Splenectomy (*%, n*)	5%	1/19	31%	26/85	**0.022**
Spleen volume >1000 mL (*%, n*)	69%	9/13	51%	23/45	0.346
Thrombocytopenia (*%, n*)^a^	47%	9/19	71%	60/85	0.064
Anemia (*%, n*)^b^	6%	1/18	46%	39/85	**0.001**
Bone complications (*%, n*)^c^	0%	0/19	33%	28/85	**0.002**
Fat fraction <23% (*%, n*)^d^	36%	5/14	48%	25/51	0.547
Decreased DLCO (*%, n*)^e^	67%	12/18	37%	19/51	0.052

*Note*: The bold values are printed in bold because they have *p*‐values < 0.05 and are therefore considered significant.

Abbreviations: ASMD, acid sphingomyelinase deficiency; DLCO, diffusion capacity of the lungs for carbon monoxide; MN, multiples of normal.

^a^ Local reference ranges for definition of thrombocytopenia were used.

^b^ Local reference ranges for definition of anemia were used.

^c^ Bone complications comprised avascular necrosis, bone crisis, pathological fracture or vertebral collapse.

^d^ Fat fraction measured with quantitative chemical shift imaging.

^e^ Decreased DLCO is defined as less than 80% of predicted based on sex, age, and length.

**TABLE 2:** Markers of disease in all patients.

			ASMD			Gaucher			
	Unit	Ref range	*n* patients	Median	Range	*n* patients	Median	Range	*p*‐value
Chitotriosidase	nmol/mL.uur	17–235	14	1693	326–6620	74	30 940	513–201 352	**<0.001**
CCL18	ng/mL	15–74	8	469	104–1050	27	948	303–1878	**0.003**
Spleen volume	mL		13	1126	550–1995	45	960	268–5358	0.551
Liver volume	ml		14	2372	1718–3465	56	2269	924–5814	0.834
Platelets	10^9^/L	150–400	19	154	86–484	85	102	16–726	**0.007**
Hemoglobin	mmol/L	♀ 7.5–10.0	18	9.0	7.0–10.4	85	7.8	5–10.4	**<0.001**
		♂ 8.5–10.5							
Leucocytes	10^9^/L	4.0–10.5	19	6.7	3.9–14.6	85	5.1	0.8–23.5	0.111
Fat fraction	%	>23	14	29.5	12–50	51	24	6–62	0.228
BMB score		‐	14	7	4–10	37	10	2–16	**0.007**
Diffusion capacity for carbon monoxide	% of predicted	80–120	18	73	26–104	51	85	54–126	**0.029**

*Note*: The bold values are printed in bold because they have *p*‐values < 0.05 and are therefore considered significant.

Abbreviations: ASMD, acid sphingomyelinase deficiency; CCL18, chemokine C–C motif ligand 18; BMB score, bone marrow burden score; DLCO, diffusion capacity of the lungs for carbon monoxide.

Median plasma values of chitotriosidase activity and CCL18 were lower in ASMD patients than in GD1 patients. Two ASMD patients and two GD1 patients were homozygous for the 24‐bp duplication in the *CHIT1* gene and were excluded from analysis. Five ASMD patients and 25 GD1 patients were heterozygous, for them chitotriosidase activity levels were doubled. Median chitotriosidase activity was 1693 nmol/mL.h (range 326–6620) in ASMD patients and 30 940 nmol/mL.h (range 513–201 352) in GD1 patients (*p* < 0.001). Median CCL18 plasma levels were 469 ng/mL (range 104–1050) in ASMD patients and 948 ng/mL (range 303–1878) in GD1 (*p* = 0.003). In the subgroup of non‐splenectomized patients (ASMD *n* = 18, GD1 *n* = 59) these differences were present as well (see supplemental table for an overview of the markers in the subgroup of non‐splenectomized patients).

Spleen and liver volumes did not differ between the two groups. Median spleen volume was 1126 mL (range 550–1995) in ASMD patients (data available for 13 patients) and 960 mL (range 268–5358) in GD1 patients (data available for 45 patients, *p* = 0.551). Median liver volume was 2372 mL (range 1718–3465) in ASMD (data available for 14 patients) and 2269 mL (range 924–5814) in GD1 (data available for 57 patients, *p* = 0.834). Organ volumes in multiples of normal (i.e., based on patients' weight) did not differ between both diseases either.

Plasma levels of platelets and hemoglobin were higher in ASMD patients than in GD1 patients. Median platelet levels were 154 10^9^/L (range 86–484) in ASMD and 102 10^9^/L (range 16–726) in GD1 (*p* = 0.007). Median hemoglobin levels were 9.0 mmol/L (range 7.0–10.4) in ASMD and 7.8 mmol/L (range 5.0–10.4) in GD1 (*p* <0.001). In non‐splenectomized patients, these differences were more pronounced (see supplemental table). Reference values of hemoglobin are different for men and women, within subsets of male and female patients the differences between ASMD and GD1 remained (data not shown). Anemia was present in 6% of ASMD patients compared to 46% of GD1 patients (*p* = 0.001). Thrombocytopenia was present in 47% of ASMD patients compared to 71% of GD1 patients (*p* = 0.064).

**FIGURE 3:**
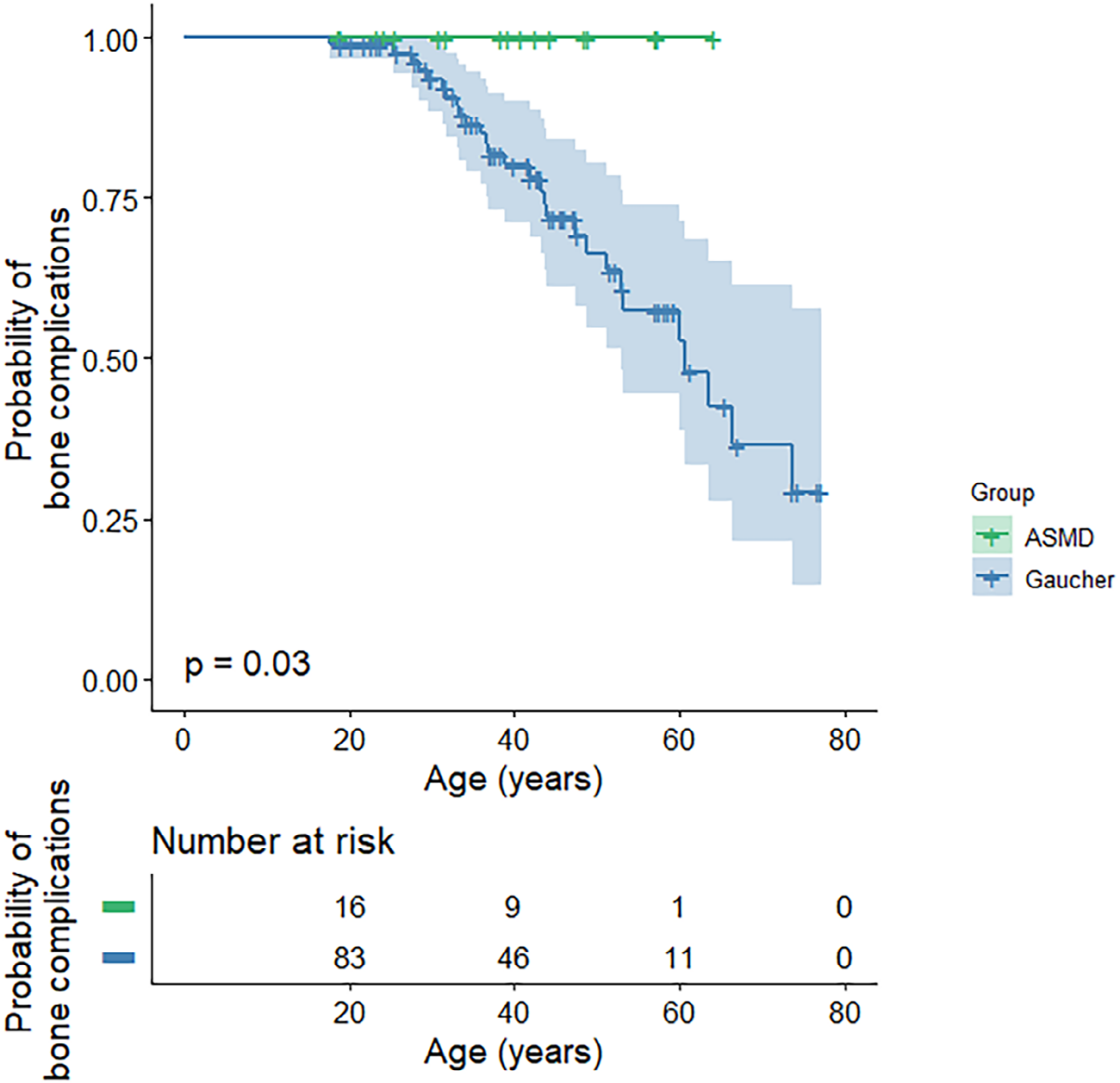
Probability of bone complications plotted against age for ASMD and Gaucher patients. ASMD, acid sphingomyelinase deficiency.

Fat fraction (Ff) values measured with QCSI were available for 14 ASMD patients and 51 GD1 patients. Ff was similar for both groups with medians of 29.5% (range 12–50) in ASMD and 24% (range 6–62) in GD1 (*p* = 0.228). The percentage of patients with Ff values under 23% (previously suggested as the threshold for increased risk of bone complications in GD1^18^) was similar as well: 36% of the ASMD patients had Ff values below 23%, which was the case for 48% of the GD1 patients (*p* = 0.547). Median BMB scores were 7 (range 4–10) for ASMD patients and 10 (range 2–16) for GD1 patients (*p* = 0.007). No bone complications were reported for ASMD patients, while in 33% of the GD1 patients bone complications (i.e., avascular necrosis, bone crisis, pathological fracture, or vertebral collapse) were reported (*p* = 0.002). In Figure 3 the probability of bone complications is plotted against age for ASMD and GD1 patients (*p* = 0.030, log rank test).

Pulmonary involvement was more profound in ASMD. It should be noted that pulmonary function tests were standardly performed in GD1 patients between 2000 and 2001 and later on indication, therefore data were available for 51 out of 85 GD1 patients. Median DLCO was 73% of predicted (range 26–104) in ASMD patients and 85% of predicted (range 54–126) in GD1 patients (*p* = 0.029). Decreased DLCO was found in 67% of ASMD patients and in 37% of GD1 patients (*p* = 0.052).

Findings were similar in an age‐ and sex‐matched cohort: significant differences were found between ASMD and GD1 patients for chitotriosidase activity and BMB score (both lower in ASMD with a similar Ff in both diseases) and platelet count, hemoglobin levels, and leucocyte levels (all higher in ASMD, see supplemental Table 1 and 2 and supplemental Figure 1).

**FIGURE 4:**
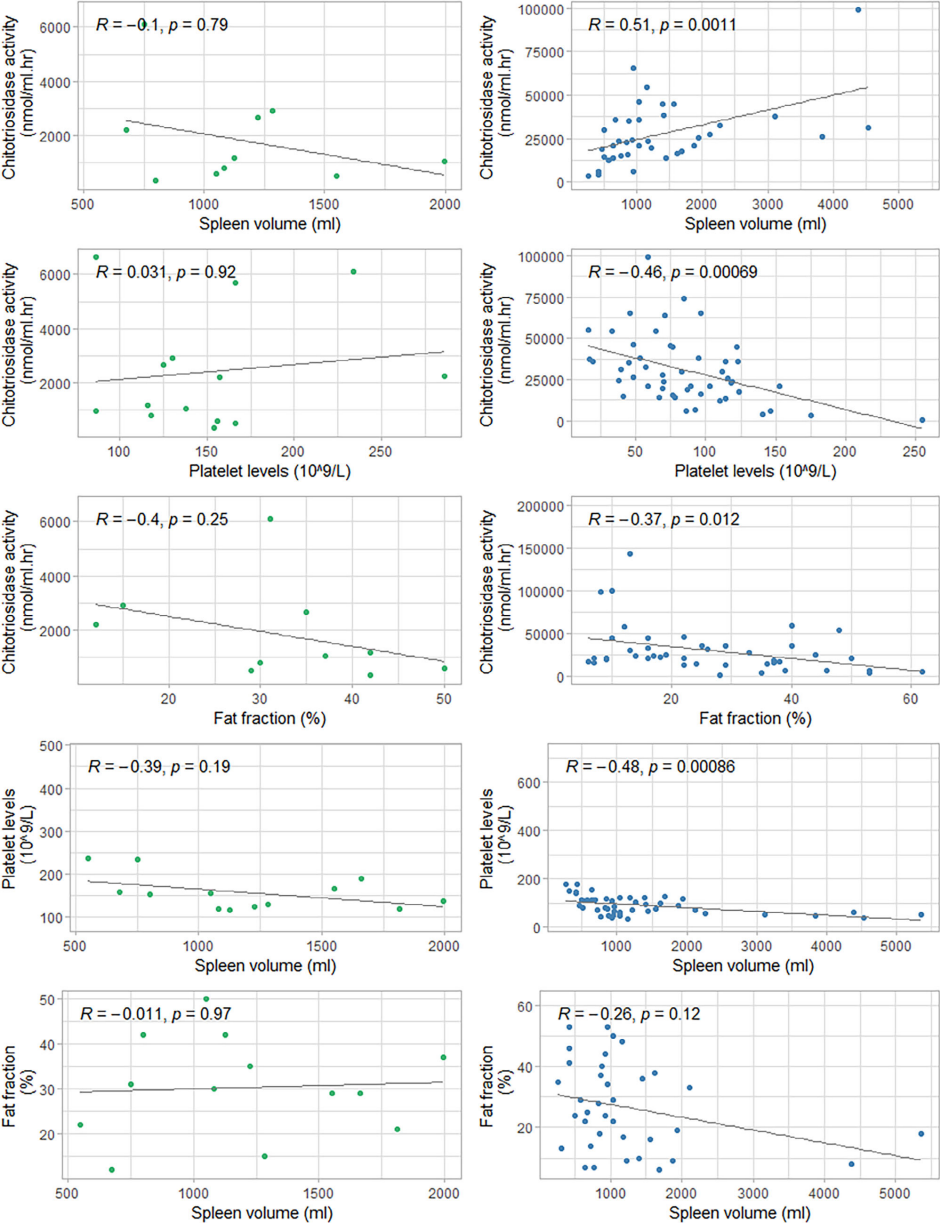
Correlations between several biomarkers in ASMD (left) and Gaucher (right). ASMD, acid sphingomyelinase deficiency.

As described previously, correlations between chitotriosidase activity and numerous markers (e.g., spleen volume, platelet count and Ff) have been established for GD1.^20^, ^21^ In Figure 3 correlations between several markers are depicted for the ASMD and GD1 cohort of this study. In this cohort of GD1 patients, correlations were present between chitotriosidase activity and spleen volume, platelet count, and Ff (respectively *R* = 0.51, *p* = 0.001, *R* = −0.46, *p* < 0.001 and *R* = −0.37, *p* = 0.012). Additionally, correlations between spleen volume and platelet count were present (*R* = −0.48, *p* < 0.001). These correlations were not found in this cohort of ASMD patients (see Figure 4).

## 4 DISCUSSION

The aim of this study was to compare cross‐sectional clinical and biochemical data of untreated adult ASMD patients with the chronic visceral subtype and untreated adult GD1 patients in order to better understand the similarities and differences between both LSDs. While several clinical manifestations of ASMD and GD1 showed overlap, clear differences were observed as well. First of all, the extend of hepatosplenomegaly was similar in both groups with significant enlargements of spleen and liver. Although a similar splenomegaly would suggest equal sequestration of blood cells in the spleen, platelets and hemoglobin plasma levels were strikingly lower in GD1 than in ASMD, which is in line with previous research.^11^, ^22^, ^23^ This would suggest a difference in the degree of compromised hematopoiesis in the bone marrow. Hence bone marrow infiltration with lipid‐laden macrophages, related to clinical bone disease, could be different in these disorders.

Indeed, bone complications were present in a third of GD1 patients while absent in this ASMD cohort. However, Ff values measured by QCSI were comparable in both groups. QCSI is a technique to assess the Ff in bone marrow. It was developed and validated as a biomarker of bone involvement in GD1 in our center and was therefore also measured in ASMD patients.^18^, ^24^ Decreased Ff in GD1 is assumed to be caused by the accumulation of storage cells in the bone marrow, thereby compromising hematopoietic cells as well as adipocytes containing triglycerides.^9^ An Ff lower than 23% was defined as a critical threshold in GD1 patients and as an independent parameter to consider to start therapy.^14^, ^18^ Validation studies showed that Ff is subject to several variables, including age, sex, increase in water fraction due to active hematopoiesis, and other factors.^14^ So, while differences in Ff could not be established, BMB scores, an MRI‐based parameter to reflect bone marrow invasion,^19^ were higher in GD1 than in ASMD and therefore reflected the differences in bone involvement more accurately.

Both ASMD and GD1 patients had elevated chitotriosidase activity and CCL18 plasma levels, but GD1 patients had significantly higher levels than ASMD patients. Studies in which chitotriosidase activity was compared between ASMD and GD1 describe similar findings of distinct elevation in GD1 as compared to ASMD.^25^, ^26^, ^27^, ^28^ This indicates a higher sphingolipid storage load in GD1 than in ASMD as was previously suggested.^29^ Moreover, in patients with the severe phenotype of Fabry disease (OMIM 301500), another LSD, chitotriosidase is mildly elevated.^30^ One study directly comparing chitotriosidase activity in these three LSDs showed that ASMD patients had higher chitotriosidase activity than classical male Fabry patients, while GD1 patients had the highest levels.^28^

In contrast, pulmonary manifestations were more common in ASMD than in GD1. This is in line with previous research showing that pulmonary manifestations are a hallmark of ASMD: more than 75% of the ASMD patients showed signs of interstitial lung disease and/or decreased DLCO in two natural history cohorts.^6^, ^11^ In GD1 patients dyspnea, interstitial lung disease and decreased DLCO have been described as well, albeit to a lesser extent and in more severe cases.^8^, ^31^

**FIGURE 5:**
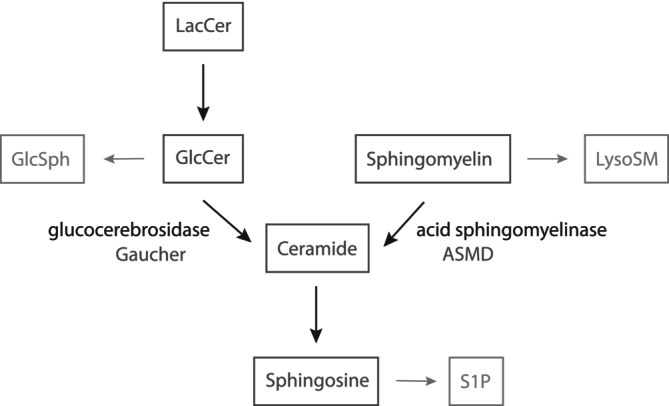
Sphingolipid metabolism surrounding sphingomyelin and glucosylceramide. GlcCer, glucosylceramide; GlcSph, glucosylsphingosine; LacCer, lactosylceramide; lysoSM, lysosphingomyelin; S1P, sphinosine‐1‐phosphate.

The difference in bone and pulmonary manifestations between ASMD and GD1 indicates contrasting local (e.g., organ specific) pathophysiological processes surrounding sphingolipid accumulation. Sphingolipids are bioactive and regulate key cell processes such as apoptosis, cell differentiation, and autophagy, but also maintain balance in these processes by reciprocal stimulation and inhibition.^32^, ^33^ Sphingomyelin and glucosylceramide are closely related sphingolipids, both can be degraded to ceramide, which in turn can be converted to sphingosine and sphingosine‐1‐phosphate (see Figure 5). Several factors might partially explain different effects of sphingomyelin and glucosylceramide accumulation (see Figure 5): (I) local effects of the storage material or storage cell, (II) local effects of derived metabolites (i.e., lysosphingomyelin and glucosylsphingosine), (III) disturbances within the metabolic pathway (e.g., depletion of substrate for formation of upstream metabolites or increased formation of downstream metabolites), (IV) secondary accumulation (e.g., cholesterol in ASMD) and (V) secondary inflammatory processes triggered by (some of) these processes. Potentially, the higher storage load and/or enhanced macrophage activation in GD1 triggers (some of) these processes to a larger extent explaining the more extinct clinical manifestations. Moreover, in ASMD more storage of sphingomyelin outside the lysosomes might be present since sphingomyelin is an important component of the plasma membrane and is present throughout the cell.^34^ This might lessen the burden of lysosomal storage and might trigger different pathophysiological processes. Lastly, these processes probably differ between tissues. In bone tissue for instance, sphingolipids are suggested to play a role in the regulation of both osteoblast and osteoclast function.^35^, ^36^ In pulmonary tissue, the ratio between ceramide and sphingosine‐1‐phosphate levels has been proposed to play a role in maintaining cell survival and vascular barrier function.^37^, ^38^

A strength of this study is the well‐defined patient cohort; all ASMD and GD1 patients are monitored in the national referral center according to the most recent scientific insights. Diagnoses are confirmed by enzyme activity measurements and mutation analyses, and biochemical markers are measured in a dedicated lab and have been studied extensively.^15^, ^16^, ^20^, ^28^, ^39^ The clinical and biochemical markers have not been validated as biomarkers for ASMD, but they were carefully reviewed by our group and others.^40^, ^41^ Nonetheless, this study has some limitations: first, a cross‐sectional study design was used since the majority of GD1 patients started therapy soon after the baseline assessments; therefore, some markers are missing and patients could not be followed over time. Most of the missing markers can be explained (e.g., chitotriosidase deficiency and some patients did not undergo MRI scans due to ineligibility or claustrophobia). Lastly, it should be mentioned that pulmonary function tests were performed in GD1 patients in the context of a clinical study during a few years. Afterward, pulmonary function tests were in some cases performed at the baseline visit of new patients, a selection bias in this group might be present.

In conclusion, bone complications, hematological abnormalities, elevation of chitotriosidase activity, and of CCL18 plasma levels are more excessive in GD1 than in ASMD. A decreased DLCO is more commonly observed in ASMD. Different secondary pathophysiological processes surrounding sphingomyelin and glucosylceramide accumulation and a higher sphingolipid load in GD1 might (partially) explain these differences. Future research could focus on elucidation of the biochemical processes surrounding sphingomyelin and glucosylceramide accumulation, potentially leading to new therapeutic targets.

## AUTHOR CONTRIBUTIONS

This study was conceptualized by EE, BS, and CH. Analysis was performed by EE. Draft preparation was done by EE, BS, and CH. All authors reviewed the manuscript.

## CONFLICT OF INTEREST STATEMENT

LvD, JA, MvdL, MM, EA, and AvK have no competing interests to declare. EE is involved in a pre‐marketing study with Sanofi Genzyme as a sub‐investigator. BS was involved in premarketing studies with Chiesi and Reneo Pharmaceuticals. CH is involved in premarketing studies with Sanofi Genzyme, Protalix, and Idorsia.

## INFORMED CONSENT STATEMENT

All procedures followed were in accordance with the ethical standards of the responsible committee on human experimentation (institutional and national) and with the Helsinki Declaration of 1975, as revised in 2000. Informed consent was obtained from all patients for being included in the study.

## Supporting information


**Data S1:** Supporting information

## Data Availability

The datasets used and/or analyzed during the current study are available from the corresponding author on reasonable request.
