# Supplementary material for: Acid sphingomyelinase deficiency and Gaucher disease in adults: Similarities and differences in two macrophage storage disorders
Source: JIMD Rep. 2024 Jul 4;65(5):330–40. doi: 10.1002/jmd2.12420 (PMC11558470; doi:10.1002/jmd2.12420)
Supplement: Supplementary file 1 — Data S1: Supporting information [file JMD2-65-330-s001.docx]

**Supplemental files**

|  | | |  |  |  |  |  |  |  |
| --- | --- | --- | --- | --- | --- | --- | --- | --- | --- |
|  |  |  | ASMD | | | Gaucher | | |  |
|  | unit | ref range | n patients | median | range | n patients | median | range | p-value |
| Chitotriosidase | nmol/ml.hr | 17-235 | 14 | 1693 | 326-6620 | 50 | 25798 | 513-98992 | **<0.001** |
| CCL18 | ng/ml | 15-74 | 7 | 440 | 104-1050 | 16 | 1034 | 303-1754 | **0.010** |
| Spleen volume | ml | - | 13 | 1126 | 550-1995 | 45 | 960 | 268-5358 | 0.551 |
| Liver volume | ml | - | 13 | 2411 | 1718-3465 | 45 | 2226 | 924-4201 | 0.428 |
| Platelets | 10^9^/L | 150-400 | 18 | 146 | 86-286 | 59 | 78 | 16-255 | **<0.001** |
| Hemoglobin | mmol/l | ♀ 7.5-10.0 | 17 | 8.9 | 7-10.1 | 59 | 7.8 | 5-10.4 | **<0.001** |
|  |  | ♂ 8.5-10.5 |  |  |  |  |  |  |  |
| Leucocytes | 10^9^/L | 4.0-10.5 | 18 | 6.5 | 3.9-13.4 | 59 | 4.2 | 0.8-18. 1 | **<0.001** |
| Fat fraction | % | >23 | 13 | 30 | 12-50 | 37 | 26.5 | 6-53 | 0.363 |
| BMB score | - | - | 13 | 7 | 4-10 | 33 | 10 | 2-16 | **0.017** |
| DLC | % of predicted | 80-120 | 17 | 71 | 26-103 | 35 | 88 | 54-126 | **0.004** |

**Supplemental table 1. Markers of disease in non-splenectomized cohort.** ASMD: acid sphingomyelinase deficiency, CCL18: chemokine C-C motif ligand 18, BMB score: bone marrow burden score, DLCO: diffusion capacity of the lungs for carbon monoxide, p-values indicate differences in medians between disease groups.

|  | |  |  |  |  |  |  |  |  |
| --- | --- | --- | --- | --- | --- | --- | --- | --- | --- |
|  |  |  | ASMD | | | Gaucher | | |  |
|  | unit | ref range | n patients | median | range | n patients | median | range | p-value |
| Chitotriosidase | nmol/ml.hr | 17-235 | 12 | 1101 | 326-6092 | 12 | 20524 | 2960-98992 | **<0.001** |
| Spleen volume | ml | - | 13 | 1126 | 550-1995 | 13 | 1042 | 641-5358 | 0.918 |
| Liver volume | ml | - | 13 | 2411 | 1718-3465 | 13 | 2486 | 1598-3617 | 0.579 |
| Platelets | 10^9^/L | 150-400 | 13 | 154 | 116-237 | 13 | 76 | 38-122 | **<0.001** |
| Hemoglobin | 10^9^/L | ♀ 7.5-10.0 | 13 | 9.0 | 7.8-10.1 | 13 | 8.3 | 6.8-9.7 | **0.048** |
|  |  | ♂ 8.5-10.5 |  |  |  |  |  |  |  |
| Leucocytes | mmol/l | 4.0-10.5 | 13 | 6.7 | 3.9-13.4 | 13 | 4.0 | 3.4-9.2 | **0.001** |
| Fat fraction | 10^9^/L | >23 | 13 | 30 | 12-50 | 13 | 22 | 8-53 | 0.342 |
| BMB score | - | - | 11 | 7 | 4-10 | 11 | 11 | 6-13 | **0.018** |
| DLCO | % of predicted | 80-120 | 7 | 69 | 26-98 | 7 | 104 | 83-126 | **0.007** |

**Supplemental table 2. Markers of disease in matched cohort.** CCL18 is omitted because of too many missing datapoints. ASMD: acid sphingomyelinase deficiency, CCL18: chemokine C-C motif ligand 18, BMB score: bone marrow burden score, DLCO: diffusion capacity of the lungs for carbon monoxide, p-values indicate differences in medians between disease groups.


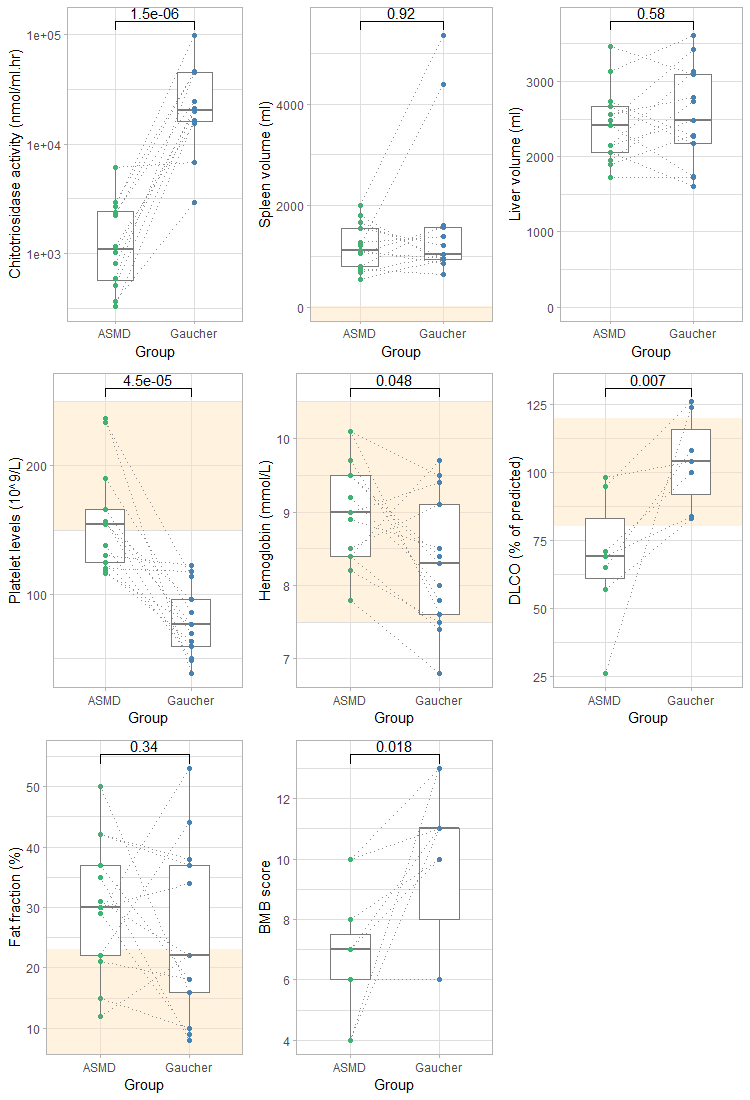


**Supplemental figure 1. Markers of disease in matched cohort.** Boxes show median and interquartile ranges, yellow squares indicate reference ranges if available, dotted lines connect two age- and sex-matched patients. CCL18 is omitted because of too many missing datapoints. ASMD: acid sphingomyelinase deficiency, BMB score: bone marrow burden score.
